# Treatment of Down Syndrome-Associated Arthritis with JAK Inhibition

**DOI:** 10.1155/2022/4889102

**Published:** 2022-07-16

**Authors:** Jordan T. Jones

**Affiliations:** ^1^Department of Pediatrics, Children's Mercy Kansas City, Kansas, MO, USA; ^2^University of Missouri-Kansas City School of Medicine, Kansas, MO, USA; ^3^University of Kansas School of Medicine, Kansas, KS, USA

## Abstract

Down syndrome (DS) results from a trisomy of chromosome 21, which causes immune dysregulation that leads to hyperactivation of interferon and Janus kinase (JAK) signaling. This results in complex medical abnormalities in the immune system and an increase in autoimmune and autoinflammatory conditions such as down syndrome-associated arthritis (DA). DA is an aggressive, destructive, inflammatory arthritis that is easily misdiagnosed and difficult to treat. Treatment commonly includes immunosuppressive therapy, but these are often associated with adverse effects and ineffectiveness. This case report outlines a 6-year-old male with DA that was successfully treated with the JAK inhibitor tofacitinib. Due to the aggressive nature of DA and poor response to many immunosuppressive therapies, this case report was created to increase awareness of JAK inhibition as an effective, well-tolerated treatment for DA.

## 1. Introduction

Down syndrome (DS) is one of the most common birth defects in the United States [1] and the most common genomic disorder that results from a trisomy of human chromosome 21 [2]. The trisomy 21 results in an increased prevalence of hypothyroidism, congenital heart disease, hematologic disorders, and autoimmune disease such as celiac disease, diabetes mellitus, and down syndrome-associated arthritis (DA) [3]. It has been established that there is immune dysregulation in those with DS that contributes to elevated levels of the tumor necrosis factor (TNF) and interferon (IFN) [4] as the trisomy 21 causes hyperactivation of IFN and Janus kinase (JAK) signaling [5, 6] and may be responsible for the cause of DA. Most children with DA present with polyarticular (five or more joints with arthritis at presentation), rheumatoid factor (RF), and antinuclear antibody (ANA) negative disease that is aggressive with bone and joint damage at presentation. There is also a long delay in diagnosis due to lack of screening guidance and misdiagnosis due to common mechanical issues seen in those with DS such as pes planus, hypermobility, and hypotonia [3]. Once diagnosed, and despite aggressive immunosuppressive therapy, the disease burden remains high, and therapeutic challenges are common due to an increase in adverse effects and ineffectiveness of therapy [7]. Synthetic and biologic disease modifying antirheumatic drugs (DMARDs) have not been as successful as a therapeutic option for DA compared to other forms of arthritis due to poor disease control and adverse effects [3]. With the hyperactivation of IFN and JAK signaling seen in DS, JAK inhibition is an appealing option for disease control. This report describes the first reported case of a pediatric patient with DA who was successfully treated to clinically inactive disease with the JAK inhibitor tofacitinib.

## 2. Case Presentation

A 6-year-old male with trisomy 21 (meiotic nondisjunction), hypothyroidism (with negative thyroid peroxidase and thyroid globulin antibodies), and a history of frequent bacterial, viral, and topical fungal infections with hypogammaglobulinemia requiring IVIg was seen in the rheumatology clinic in July of 2017 for a 12-month history of bilateral foot and ankle pain. There was right ankle swelling and limitation that had been present for the previous three months, which was thought to be correlated to an increase in activity over that time frame with starting school and a new dance class. The right ankle swelling fluctuated in severity but never completely resolved, and there were 20 minutes of morning stiffness daily. The swelling and limitation did improve with ibuprofen (10 mg/kg every 6 hours as needed) but never resolved. On physical exam, the patient was noted to have swelling and limitation of bilateral ankles with antalgic gait along with generalized joint hypermobility (Beighton score of 8/9) and bilateral pes planus. His initial laboratory evaluation revealed a normal C-reactive protein (CRP; <0.5 mg/dL), erythrocyte sedimentation rate (ESR; 8 mm/h), complete blood count (without any cytosis or cytopenia), negative ANA, RF, and human leukocyte antigen B-27. He had low immunoglobulin G (602 mg/dL), immunoglobulin M (44 mg/dL), and normal immunoglobulin A (81 mg/dL). An ultrasound of bilateral ankles and feet showed the fluid surrounding the bilateral extensor digitorum longus, left peroneal brevis and longus tendons, and left extensor hallucis longus tendon consistent with tenosynovitis. He was started on naproxen (10 mg/kg twice daily), and after eight weeks, he showed minimal improvement in pain, swelling, and limitation. The family was hesitant to start immunosuppressive therapy due to his history of recurrent infections with hypogammaglobulinemia, and so he was switched to meloxicam (0.25 mg/kg daily). After an eight-week course, there was no improvement in ankle and foot pain and swelling, and he continued to have antalgic gait. His morning stiffness persisted and increased to 30 minutes daily. He also developed swelling and discomfort in his bilateral second and third metacarpophalangeal joints. His family remained hesitant about immunosuppression but was interested in additional therapy, and hydroxychloroquine (3.5 mg/kg daily) was added. The combination of meloxicam and hydroxychloroquine did result in mild improvement in ankle, foot, and hand swelling and limitation over the next five months; however, none of the symptoms resolved. There were plans to start IV abatacept for continued evidence of active arthritis; however, the patient developed seizures and regression with oral intake, eating, toileting, and weight loss, and the family wanted to prioritize evaluation and treatment of neurodevelopmental and behavioral issues, so the patient was continued on the combination of meloxicam and hydroxychloroquine. The medications were inconsistently used due to the other health issues related to DS until April 2020 when he developed an upper respiratory infection with fever and had worsened joint pain, limitation, and swelling in bilateral ankles, feet, and metacarpophalangeal joints. In addition, he developed swelling and limitation in the bilateral knees. After resolution of the febrile illness he continued with pain, swelling, limitation, and antalgic gait. He did not want to engage in activities he usually enjoyed and would avoid physical activities as well. After his febrile illness resolved, laboratory tests showed normal platelets (429 × 10^3^/mcL) and hemoglobin (13.3 gm/dL) with low white blood cells (4.1 × 10^3^/mcL), elevated ESR (18 mm/h), and normal CRP (<0.5 mg/dL). With the increase in inflammatory arthritis symptoms and impact on quality of life, the family decided they were ready to escalate to immunosuppressive therapy. A discussion of risks and benefits was undertaken to outline additional therapy options. We decided to avoid corticosteroids due to the common adverse effects such as weight gain that is commonly experienced in children with DS [8]. We also avoided synthetic DMARDs such as methotrexate due to the increased intolerance and adverse effects experienced in children with DS [9]. In addition, evidence shows that many patients with DA have poor response to biologic DMARDs such as anti-TNF inhibitors [7], so we looked for another option for treatment. Since trisomy 21 causes elevated levels of IFN through JAK signaling, a JAK inhibitor such as tofacitinib was determined to be an appealing possible therapeutic option. In addition, the family was worried about administration of injectable synthetic and biologic DMARDs, which can be challenging in the pediatric population. Furthermore, the patient was under investigation for autism due to behavioral changes and regression, and the family felt an injectable therapy would be difficult to administer regularly. At the time (April 2020), no JAK inhibitor was approved by the United States Food and Drug Administration (FDA) for arthritis in children, but it was under investigation in clinical trials and was approved for use in adults with inflammatory arthritis. JAK inhibition was determined to have good potential for disease control, and so tofacitinib was initiated at 2.5 mg orally, twice daily. There was a rapid improvement in joint swelling, limitation, and stiffness. There was no more morning stiffness or antalgic gait. The patient was noted to be more active with improved mood, and there was complete resolution of arthritis two months after initiation of tofacitinib. The laboratory tests (CRP and ESR) continued to be normal, and there were no adverse effects or serious infections reported since initiation approximately 28 months ago.

## 3. Discussion

Down syndrome-associated arthritis (DA) is an aggressive, destructive, inflammatory arthritis that has long delay in time from symptom onset to diagnosis that can be up to three years due to being underrecognized [9]. In addition, those with DS commonly have hypotonia, hypermobility, pes planus, and autism, which may mislead providers and further delay diagnosis. Patients with DA commonly present with bone and joint damage at presentation; however, the patient reported here was diagnosed earlier than usual as the down syndrome clinic at our institution has a brief musculoskeletal screen given to all patients seen in the DS clinic, which includes focused questions about musculoskeletal pain and limitation with positive screens that trigger additional evaluation and referral as appropriate [10]. This does provide support for regular musculoskeletal screening for patients with DS as DA has a prevalence of 10 per 1000 patients [3]; however, the optimal timing and interval of screening is unclear.

Once diagnosed, the therapeutic approach to DA has been similar to juvenile idiopathic arthritis (JIA) [11], which is the most common rheumatic disease in childhood. JIA is an autoimmune disease that results in arthritis in children, and studies have shown differences in clinical presentation and pathogenesis between DA and JIA. A study that evaluated the adaptive immune system of individuals with DA and JIA found that those with DA had expansion of IgM-only memory B cells and decreased number of transitional B cells. It was also noted that those with DA have increased proinflammatory cytokines TNF and INF compared to those with JIA. It is thought that the proinflammatory cytokine response is associated with increased T cell plasticity in those with DA [12]. There have also been findings to suggest that the synovial tissue inflammation is different between those with DA and JIA with increased infiltration of T and B cells in the inflamed synovium in DA [12]. The immune dysregulation in DA is likely in part caused by the four IFN receptors encoded on chromosome 21, which leads to hypersensitivity of IFN ligands, increased JAK signaling, and increase in proinflammatory autoimmune and autoinflammatory disease [5, 6, 13]. Evidence continues to build that shows a connection among increased IFN, JAK signaling, and inflammatory disease in those with DS. In September 2020, the JAK inhibitor tofacitinib was approved by the FDA for treatment of polyarticular JIA in children of 2 years of age and older. This approval may make it easier for those with DA to obtain this medication now, as some view DA and JIA as the same disease [11], despite growing evidence to suggest otherwise [3]. In addition, JAK inhibition has also been used to successfully treat psoriatic arthritis in an adult with DS [14] with rapid improvement in skin and joint disease and no adverse effects. This further supports JAK inhibition for inflammatory conditions seen in DS.

DA is an aggressive, destructive, inflammatory joint disease in those with DS that is poorly understood; however, as awareness of DA improves and as more individuals with DS are diagnosed, we will need better therapies and therapeutic approaches that are tailored to those with DA. The current therapeutic options for DA, which have been borrowed from JIA, come with more adverse effects and less effectiveness; however, JAK inhibition may become a preferred option for those with DA as it is oral medication, has proven effective for arthritis treatment, and targets the immune dysregulation seen in those with DS.

## Data Availability

All data used to support the findings of this study are available upon request to the corresponding author.
